# Factors influencing contraceptive decision making and use among young adolescents in urban Lilongwe, Malawi: a qualitative study

**DOI:** 10.1186/s12978-021-01259-9

**Published:** 2021-10-18

**Authors:** Gift Mtawali Dombola, Wanangwa Chimwaza Manda, Effie Chipeta

**Affiliations:** 1 Department of Health Systems and Policy, College of Medicine, Private Bag 360, Blantyre, Malawi; 2 The Centre for Reproductive Health, College of Medicine, Private Bag 360, Blantyre, Malawi

**Keywords:** Young adolescents, Contraceptives, Decision making, and use

## Abstract

**Background:**

The prevalence of teenage pregnancies in Malawi is 29%. About 25% of those are married while 30% are unmarried adolescents (15–19 years old) who use contraceptives. Data on contraceptive use has focused on older adolescents (15–19 years old) leaving out the young adolescents (10–14 years old). This study assessed factors that influence contraceptive decision-making and use among young adolescents aged 10–14 years.

**Methods:**

This was a qualitative study that used the Theory of Reasoned Action (TRA) model to understand the processes that influence contraceptive decision-making among young adolescents (10–14 years old) in urban Lilongwe. The study was conducted in six youth health-friendly service centers and 12 youth clubs. Two focus group discussions and 26 in-depth interviews were conducted among sexually active in and out of school young adolescents and key informants. The results are organized into themes identified during the analysis.

**Results:**

Results showed that contraceptive decision-making is influenced by social factors (individual, interpersonal, society) and adolescents’ perceptions regarding hormonal contraceptives. There is also a disconnect between Education and Adolescent Sexual and Reproductive Health policies.

**Conclusion:**

The findings suggest that interventions that scale up contraceptive use need male and female involvement in decision making. Addressing myths around contraceptives, and harmonization of Education and Sexual and Reproductive Health policies in the country would motivate adolescents to use contraceptives.

## Plain language summary

There is limited data on modern family planning method use of young adolescents aged 10–14 years compared to older adolescents aged 15–19 years. The study assessed factors that influence modern family planning method decision-making and use among young adolescents aged 10–14 years.

The Theory of Reasoned Action model was applied to understand the processes that influence modern family planning method decision-making and use among young adolescents. The study was conducted in 6 youth health-friendly services clinics and 12 youth clubs where adolescents access contraceptives. We conducted 2 focus group discussions and 26 in-depth interviews. The study involved sexually active, in and out of school young adolescents and influential decision-makers in reproductive health. The results are presented in a narrative format using themes identified during analysis in line with study objectives.

The study discovered that modern family planning method decision-making is influenced by social factors; hormonal side effects of modern family planning methods and improper linkage between Sexual and Reproductive Health Rights and Education policies.

The findings suggest the need to empower girls in decision-making. Address myths surrounding modern family planning methods. Then harmonize Sexual and Reproductive Health Rights and Education Policies in the country.

## Background

Globally, the population of adolescents is estimated at 1.2 billion, and a half are very young adolescents [1]. Adolescents are categorized into two groups; young adolescents (10–14 years old) and older adolescents (15–19 years old) [2]. In 2016, 545 million very young adolescents were from developing countries with 63% from Asia, 26% from Africa, 10% from Latin America, and the Caribbean [1]. Malawi, one of the developing countries in sub-Saharan Africa, 40% of the total population are adolescents aged below 15 years [3].

Studies conducted in developing and developed countries indicate that most adolescents face sexual and reproductive health problems [2]. Some of the factors that contribute to these problems include early sexual debut and early maturity where 17–31% of girls aged 12–14 years in some parts of Africa including Malawi experience menstruation [4]. Peer pressure, inadequate parental support, and increased curiosity to engage in intimate relationships as their bodies develop from childhood to adulthood also contribute to risky sexual behaviors [5]. In addition to that, the rapid growth of technology where adolescents spend more time on social network sites interacting with friends who have become the main source of information [5]^8^. Along the way, they engage in intimate relationships that lead to risky sexual behaviors such as unprotected sexual intercourse [5–7]. Unprotected sex puts them at higher risk of getting unintended pregnancies, and sexually transmitted infections including human immunodeficiency virus (HIV) [7]. Early sexual debut in this study is defined as having sex before or at the age of 14 that contributes to early marriages, sexually transmitted infections including cervical cancer in young adolescent girls [7]. In Caribbean countries, early sexual debut is at 37.2% and 16.9% in boys and girls respectively [6]. In Southern, middle, and west Africa, the early sexual debut is quite similar in both sexes and ranges from 18 to 25% while in eastern Africa early sexual debut is low between 13 to 15% of women having sex before the age of 15 years [8]. In Malawi, 13% of females and 22% of males had their first sexual intercourse before 15 years of age [3]^9^. And 7% of adolescent girls aged 15–19 years old get pregnant before 15 years of age[3].

Global estimates show that at the age of 17, adolescents girls are 5 times more likely to conceive if they do not use contraceptives compared to those who use contraceptives at their first sexual intercourse [9]. Babies born to adolescent mothers are likely to be premature and of low birth weight [10]. In addition, births among adolescent girls are often accompanied by obstetric complications such as fistula, uterine rupture, postpartum hemorrhage, and preeclampsia [1]. These complications contribute to high maternal morbidity and mortality and negative social-economic outcomes among adolescent girls [1, 11, 12]. Despite high morbidity and mortality, this age group faces several challenges when accessing health services such as health providers` attitudes, long distances, and limited resources. In Malawi, 43.67% of young people in Salima, Dedza, and Mangochi districts are unable to access youth-friendly health services because some schools are not linked to donor supported programs like Joint Program for Girls Education (JPGE) [13].

Estimates from low and middle-income countries show that 777,000 births occurred among adolescents aged below 15 years in 2016, with 450,660 (58%) of births occurring in Africa [1].

In Malawi, the prevalence of teenage pregnancy is at 29% [3] which has led to increased school drop-out whereby 25% of girls do not finish primary school education [9]. Teenage pregnancy also leads to early marriages, depression, and suicidal attempts [13–15]. In addition, early childbearing leads to an increased rate of dependency, poverty, and illiteracy in the community because young mothers do not have the resources enabling them to fully take on the roles and responsibilities of taking care of the child [9]. Given these problems, contraception among this age group is important. Contraceptives prevent unintended pregnancies [16]. There is evidence that contraceptive use has risen among women of childbearing age in many countries [10]. However, there was only a modest rise of 4.9% between 2008 and 2015 in Africa [10]. Unmet need for contraception among women who want to delay or avoid pregnancy exists. Availability of contraceptives and poor quality of services offered to young adolescents and young women in and out of school poses a challenge to reducing the unmet need for contraception in the region [10].

Literature from Africa shows that young women, including adolescents, have limited access to contraceptives. This is reflected in the low use of contraception among adolescents in sub-Saharan Africa with contraceptive prevalence among this segment of the population ranging between 26 and 64% [4]. Like any other country, despite numerous interventions to address teenage pregnancy, in Malawi 25% of married and 30% of unmarried sexually active female adolescents (15–19 years) use contraception and most of these data is from demographic health surveys [17].

Interventions to empower adolescents to use contraceptives focus on older adolescents aged 15–19 years and leave out young adolescents aged 10–14 years. Therefore, there is very limited evidence on contraceptive use among sexually active adolescents aged 10–14 years given that demographic surveys usually do not include those below 15 years of age. The data on sexually active adolescents is prone to recall bias that might not give the true reflection of the situation since questions are asked retrospectively to those 15 years or older. Further to that, the data does not include behavioral questions that can be used to explore contraceptive decision-making. As a result, there is limited understanding of contraceptive decision-making processes and use among sexually active adolescents below the age of 15 years that can inform interventions to improve reproductive health outcomes among this age group hence this study.

**Fig. 1:**
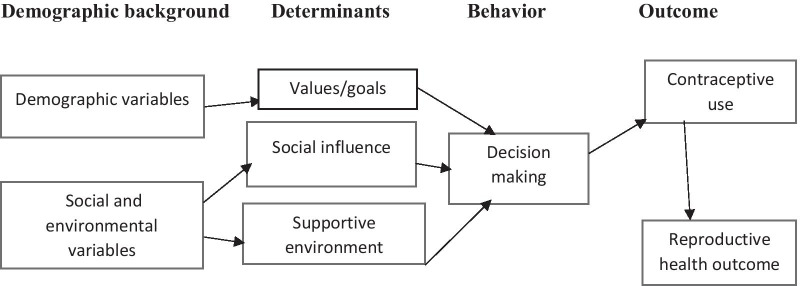
Conceptual model

The conceptual model underpinning this study has been developed from the Theory of Reasoned Action (TRA). The TRA describes the correlation between attitudes and behaviors towards human action and the study looked at the perception of adolescents towards contraceptive decision making that leads to behavior change. It posits that social factors and beliefs contribute to behavior change [18] The conceptual model incorporates determinants (values, social influence, and supportive environment), demographic background variables (age, sex, access, availability), and social and environmental variables [19, 20]. Figure 1 below is the conceptual model with details on how determinants and demographic background affect decision making.

The study used this model because decision-making is defined as a process where several considerations are reviewed before concluding [20]. In the study context, it is important to understand the decision maker’s goal or value, knowledge of contraceptives, and preferences regarding whether to initiate contraception or not. The process has multiple influences on health behaviors and outcomes. The process operates at individual, interpersonal, organizational, and societal levels and is influenced by the environment [21].

## Methods

The current cross-sectional study used a qualitative-based design (in-depth interviews and focus group discussions) and aimed to understand contraceptive decision-making and use among young adolescents aged 10–14 years. The study involved sexually active, in and out of school unmarried adolescents and key informants who were involved in decision-making regarding reproductive health.

### Study setting

It was conducted in the central region of Malawi, in urban Lilongwe. Reports indicate that urban Lilongwe is often neglected by most non-governmental organizations in implementing reproductive health interventions [22]. Most of the adolescent programs are donor-supported who have their preference and most of them prefer vulnerable adolescents in rural areas than urban [23]. The population of young adolescents (10–14 years) in Lilongwe District was 197,305 in 2018 while contraceptive prevalence among youths (10–24 years) was 33% [3]. The study was conducted in six Youth Friendly Health Service (YFHS) centers and 12 Youth Clubs (YCs) because these centers provide contraceptives where adolescents and youth aged 10–24 years can access them with minimal challenges.

### Study population

The study targeted sexually active young adolescents aged 10–14 years. In addition, key informants such as District Youth Coordinators who have direct contact with the adolescents in urban Lilongwe were involved. Plan International Representatives, the non-governmental organization (NGO) in Lilongwe that has programs that target adolescents and young mothers. During data collection Plan International was the only NGO working on adolescents in Urban Lilongwe. Community Leaders, Teachers, Religious Leaders, and Influential Community Members were included to triangulate their perspectives with those of young adolescents. Written informed consents were obtained from key informants and assents were obtained from eligible adolescents after parental/guardian permission before participation. Participants were allowed to withdraw from the study at any point if they felt uncomfortable. Table 1 below, describes the characteristics of the participants interviewed.

**Table 1:** Social demographic characteristics of participants who participated in the in-depth interviews

In-depth interview category	Sex	Number of participants	
Adolescents			
In-school	Female	6	
Out-of-school	Female	4	
In-school	Male	4	
Out-of-school	Male	4	
Total		18	
Adolescents age			
10 Years		2	
11 Years		4	
12 Years		4	
13 Years		5	
14 Years		3	
Total		18	
Adolescents sexual relationship			
≤ 12 months	Female	2	
≥ 12 months	Female	6	
≤ 12 months	Male	4	
≥ 12 months	Male	6	
Total		18	
*Key informants*	*Sex*	*Occupation*	*Total*
Acquired secondary education	Female	Teacher	1
Acquired tertiary education	Female	Health workers	2
Acquired secondary education	Male	Church leaders	4
Acquired tertiary education	Male	AGYW manager	1
Sub-total			8
Grand total			26

### Sample size

Data were drawn from 26 interviews whereby eight were key informants and 18 adolescents within the study setting and two focus group discussions (FGDs). This included eight out of school unmarried adolescents and 10 in school unmarried adolescents from six YFHSs and 12 YCs. A simple random sampling was used to get 12 YCs from 36 YCs available in urban Lilongwe. The study involved sexually active, in and out of school unmarried adolescents and key informants who were involved in decision-making regarding reproductive health. And excluded adolescents who were not sexually active and informants who were not involved in decision-making. We defined a sexually active adolescent as a person who has had an intimate relationship with a person of the opposite sex for a period longer than two weeks at or before the age of 14 years [1].

### Data collection

Data was collected through in-depth interviews and focus group discussions. In-depth interviews took about 30–35 min per participant while FGDs took an average of 1 h 10 min. All interviews were audio-recorded. A semi-structured guide was used to understand the age at which young adolescents had their first sexual relationship, contraceptives use/method, and the processes of contraceptives decision-making. Some of the questions that were used included:

- Would adolescents like to be an advocate of modern family planning methods to sexually active young adolescents? What about yourself?
- Do adolescents discuss issues about sexuality and contraceptive use with their parents/guardians? Why?
- What do adolescents consider important before using modern family planning methods?

Tools were pre-tested and refined. Data was collected by the Principal Investigator and a research assistant for 3 months from 1st November 2018 to 30th January 2019. Key informants were interviewed individually at an agreed location upon booking an appointment with them. The researcher stopped collecting data after data saturation.

### Data analysis

Recorded interviews were transcribed verbatim. The transcripts were then translated from the local language (Chichewa) into English by the data collector. The transcripts were read several times to get the in-depth meaning of the text. Denductive and inductive codes were then developed and used to code the text. In cases where the written transcripts were not clear, the researcher referred to the audio recorder for verification. Themes were developed as they emerged during analysis surrounding the objectives of the study based on whether they reflected the factors that influenced contraceptive decision-making and use. Results are organized around themes and presented in a narrative format. Information from adolescents and key informants was triangulated as a means of validating the key findings of the study.

## Results

Three thematic areas are presented as emerged during analysis in line with the objectives of the study namely: young adolescents` perception regarding contraceptive and use, social influence on decision making, and environmental factors that influence contraceptive decision making.

### Positive perception towards the use of contraceptives

Adolescents’ perceptions of contraceptive methods varied. For instance, positive perceptions were noted regarding the use of condoms, especially from female adolescents. Condoms were mentioned as the only method that can help adolescents to prevent pregnancy and sexually transmitted infections before marriage and they should be easily accessible and distributed for free.

“…condoms are the best, because in the course of preventing pregnancy you are also preventing infections especially from unfaithful partners.” (female inschool).

### Negative perception towards the use of contraceptives

In male FGD, some participants expressed dislike for use of other contraceptive methods. The general opinion was that contraceptives do not protect against sexually transmitted infections (STIs) but rather encourage promiscuity.

“…. When girls are given contraceptives, they will be sleeping with everyone hence chances of getting sexually transmitted infections will be high.” (Male FGD).

### Side effects and aspirations

Most of the adolescents believed that contraceptives are meant for older people and not young adolescents. This view towards contraceptive use was mostly reported by female adolescents. Side effects of family planning methods were identified as the main barrier to contraceptive use hence influencing their decision to use modern contraceptive methods. In a female FGD, participants mentioned side effects of contraceptives, injectable and implants lead to missing of menses and heavy bleeding respectively hence leading to failure to adopt them.

“…. a lady should be menstruating monthly, that means she is healthy but these family planning methods I hear that you do not have your menses. And I think implants are bad because when you are young they affect your menses, sometimes you skip some months without a period and sometimes you have menses the whole month.”(Female FGD).

### Beliefs and aspirations towards contraceptives use

There was a general agreement among participants that, unlike condoms, other forms of contraceptives (family planning methods) are for married couples. They reported that they would not decide to use contraceptives, especially long-term reversible contraceptives (LARC) like implants, until marriage. Proof of fertility was mentioned as a reason for delaying contraceptive use until marriage. However, they were eager to know how best to prevent unintended pregnancy while at school without using hormonal contraceptives.

“…family planning methods are used by married couples and adolescents to prevent pregnancy by use of condoms or else abstain from having sex. That is what we are taught in youth clubs and schools respectively”. (Males adolescents in school).

### Fears and concerns regarding contraceptive use

Most of the female adolescents expressed fears about the use of hormonal contraceptive methods. The most cited example was the fear that contraceptive use might cause cancer. These fears affect contraceptive decision-making among adolescents. Furthermore, the word ‘family planning’ was viewed as a barrier to contraceptive use among unmarried adolescents. For adolescents, family planning means that contraceptives are intended for people who have families.

“...*These things are used by married people after a proper wedding. Adolescents like us in Schools we need to finish school first. Some of the pills we hear cause cancer and You fail to conceive later in life.” (Female FGD)*

### Lack of information

Participants in key informant interviews reported a lack of information on contraceptive methods as a barrier to contraceptive use among adolescents. Key informants stated that health providers do not give comprehensive reproductive health information to adolescents. And proceeded with an example, that health providers do not give information on how contraceptives work and their side effects, and on how adolescents should respond to their body demands as they develop from childhood to adulthood. They further indicated that adolescents need the information to make an informed decision about their future and give them the right information to help clarify their fears and concerns which results in a lack of motivation to use contraceptives.

“…. The problem is that family planning methods are different and there is a need for detailed information before use. For example, when I went to the University of North Carolina (UNC) Project and they were explaining about female cup condoms that protect HIV. It was found that when they give them to adolescents, they tested HIV positive. Which means that adolescents did not skillfully use these methods because the instructions were not clear to them.” (Female key informant).

### Social influences on contraceptive decision making

The majority of participants in the male and female FGDs were of the view that the duration in a relationship was a key determinant on whether to use a contraceptive method or not. Adolescents in long-term relationships were more likely to decide to use protection than their counterparts in short-term relations. Apart from this, a partner’s faithfulness was also cited as a barrier to decision-making regarding contraception. In cases where a male adolescent discovers that a partner has multiple sexual partners, the decision to use a condom is inevitable.

“… When you have been in a relationship for so long you would not let your partner drop school just because of a pregnancy. Therefore, you ensure to use a condom to prevent pregnancy. You care about each other more than in short-term relationships.” (Male adolescent in school).

“…. In a relationship, one would suggest using a contraceptive (method) to prevent pregnancy but that is determined by the availability of a method (condom) and how long they have been in a relationship. A girl is easily known that she has another boyfriend, and if you have such type of a girlfriend, having sex without a condom you can easily get syphilis.” (Male adolescent in school).

### Peer pressure towards contraceptive use

Most male participants showed positive attitudes towards the use of contraceptives. However, there were concerns regarding friends` influence on decision-making regarding contraception. In a male FGD, participants mentioned boys discussing their experiences on contraceptives and helping each other to make a decision.

Participants further stated that boys lead in making decisions around contraceptive use. Girls usually have no much say in the relationship when it comes to sex.

“…. when you are in a relationship, boys usually lead in decision making the same as in marriage. That is why girls believe that because of love, they cannot make a decision Which will not impress a partner, you go by what he says otherwise he will dump you.” (Female adolescent out of school)

### Negative attitudes by parents/guardians towards contraceptive use

Participants in focus group discussions and key informants' interviews expressed similar views that parents perceive issues of sexuality and label contraceptives as bad things. And that they do not talk about the advantages and disadvantages of contraceptives. Some participants reported that parents are afraid of discussing such issues with their children because of cultural/religious beliefs. Adolescents raised concerns regarding parents/guardians not being open enough to discuss issues of sexuality and contraceptives in detail. They reported that parents/guardians mainly emphasize abstinence. This makes adolescents lack informed decisions regarding contraceptives.

“…Parents advise us not to impregnate a girl and not get pregnant without giving us options. When adolescents are with elder women or men they talk about abstinence as the only viable option for adolescents. Consequently, other contraceptive options are left out.” (Male FGD).

The other concern was the attitude of parents and their reaction towards adolescents. Adolescents reported that using a contraceptive method or having a method puts them at risk of being disowned by parents. However, adolescents reported that they expect much support from parents and regard them as mentors.

“….you talk of parents, aaaah that is the worst. If a girl is found with a condom, they will disown her, boys are at least better.” (Male adolescent in school).

Most of the key informants supported the view that parents do not encourage young adolescents to use contraceptives. Participants further mentioned that parents` negligence contributes to increased cases of pregnancies and school drop-outs among sexually active adolescent girls, and increased risk of complications during pregnancy and delivery. Teenage pregnancy was viewed as a huge burden on the family.

“…. If a young adolescent gets pregnant, everything stops moving. Parents (female) cannot go to work because they will be busy taking care of the adolescent at the hospital since she is young. They will not have time to go to the farm if it is rainy season. As a result, they will have no food at home and there will be severe hunger”. (Female key informant).

### Community members` perceptions regarding contraceptive use among young adolescents

It was noted that communities do not create a conducive environment regarding contraceptive use among young adolescents. Male participants were concerned about communities for not encouraging them to access contraceptives. Participants in both male and female FGDs reported that society would not allow an adolescent to exercise his or her freedom and make an independent decision.

“…. Most condoms are found in private facilities and sometimes very far that make adolescents do not think of using a method. In addition to that, if the shop seller is a parent, you will not ask for a condom to avoid embracement. They will ask you a lot of questions that you will not have answers to. For example, what is the use of the condoms you are buying? Things do not work for adolescents` favor.” (Male adolescent out of school).

### Environmental factors and contraceptive decision making

Lack of privacy and the conducive environment was cited as a barrier for adolescents to access contraceptives. This was mentioned in most of the in-depth interviews. Adolescents indicated that contraceptive methods should be easily accessible and within reach, for example in private settings like toilets. The majority were concerned about the presence of parents and other community members in most health facilities and would prefer to access contraceptive methods in schools.

Most of the participants (key informants and adolescents) supported the idea of contraceptives being available in schools for easy access among adolescents, the majority of whom are in schools. This would allow them to prevent unintended pregnancies and undesirable reproductive health outcomes and thus help them complete their studies. Apart from schools, it was also mentioned that health facilities should have special rooms to offer contraceptives to adolescents.

*“…there is need for family planning methods in schools especially in toilets so that these adolescents should have access to them without problems. In the health facilities, adolescents may face problems especially when they come across their parents/people from one village or location in the course of getting assisted*” (*female key informant*).

The lack of youth-friendly services that do not incorporate extra curriculum activities was also highlighted. Most of the adolescents aged 10–14 years raised concerns that these services are limited to older adolescents aged 15–19 and youth.

“…. Most of the time older adolescents do not welcome us in their clubs. It is better to have such clubs within school premises for easy access.” (Male in school adolescent).

### Policies around contraceptive use in schools

Participants reported the existence of policies that do not allow contraceptive methods to be distributed around the school premises, and one of them being the Education Policy. Most key informants described Education and Sexual and Reproductive Health Rights (SRHR) policies as contradicting each other, hence health providers are not free to offer contraceptives in schools. Participants reported that SRHR policy stipulates that adolescents be given information on sexual reproductive health, and family planning methods to prevent unwanted pregnancies. When the same adolescents are in school (Education Policy), things are different. No contraceptives are accessible to students within school premises. Schools and hospitals are both government institutions but policies guiding the provision of services in the institutions contradict each other.

“…. school does not allow pupils to use contraceptives, it is obvious we cannot decide the same. The Government of Malawi does not encourage the use of contraceptives in schools.” (Female FGD).

“….. in schools especially boarding schools, the information is given, yes, and in the same boarding schools, sexual relationships are there which means if the services are not given, students get pregnancies while on a school campus. If in addition to information we could provide services, we could avoid teenage pregnancies” (Female key informant).

### Advocacy/awareness around contraceptive use

Participants were asked if they would advocate for contraceptive use around school premises. The majority of those who participated in the in-depth interviews and FGDs supported advocacy for contraceptives in schools. They mentioned that this would enable adolescents to fully realize their rights to education and good health. Adolescents showed concerns about friends who were dismissed from school because they were caught with condoms. Key informants were in of the same view, mentioning that some non-governmental organizations are moving towards putting an end to such kind of practice of punishing students.

“…. We get punished, even dismissed out of school if a teacher catches you with condoms, the whole school knows about the issue. As a result, you give up on education.” (Male in-school adolescent).

“…. we are advocating right now. We have what we call School Health Days where we give out information. We do not take services because Education Policy does not allow us to take the services and yet adolescents want contraceptives”. (Male key informant).

## Discussion

Our study findings indicate that young adolescents have concerns that prevent them from deciding on whether or not to use contraceptives. Their perceptions towards contraceptives are influenced by side effects of hormonal contraceptives, cultural beliefs, and misconceptions about methods. Social and environmental factors, peer pressure, parents` attitudes, and community members shape adolescents’ actions towards contraceptive use. As the theory of reasoned action suggests, adolescents will only have an intention to use contraceptives if there is a motivation. In addition, positive attitudes and subjective norms surrounding the action towards use.

Mardi et al. [23] conducted a similar study in Iran and found that side effects of contraceptives such as missing menses and the need to prove fertility were among the issues that demotivate young adolescents from using contraception. Some cultures in Malawi believe that a woman is called a woman when she sees her menses every month. When adolescents do not have menses as a result of using contraceptives as it is common with hormonal methods, this negatively influences their perceptions altogether [25]. They would, therefore, first give birth before starting using hormonal methods or use condoms which are hormonal-free and provide dual protection.

Evidence of lack of parental support on adolescents` contraceptive use has been noted, the study also showed that young adolescents consider parents as mentors. They would prefer to get information from parents who use local languages compared to the use of medical words or professional terminologies. For example, words that looked difficult to them were family planning methods and contraceptives. These words meant that contraceptives are only for married couples. Some studies show that adolescents are usually more concerned about pregnancy than HIV infection and using terms such as pregnancy prevention could change their perception regarding contraception [5, 21, 24]. These have implications on the delivery of sexual and reproductive health (SRH) information to adolescents [25]. Consequently, advocating for use of adolescent-friendly terms in disseminating messages for preventing unwanted pregnancies and /or contraceptive use among adolescents. Considering that participants showed interest in using condoms in this study, clear, accurate, and timely information would motivate adolescents to use contraceptives [22]. In addition, enhancing outreach clinics targeting adolescents especially in hard-to-reach areas.

This study discovered that some students lose their place at school or get heavy punishment if they are caught with contraceptives. In addition, parents disown their children for having condoms. These actions from teachers and parents increase the risk of getting early and unwanted pregnancies among young adolescent girls. In contrast, a systematic review conducted in 2017 in America on the effects of school-based condom availability programs (CAPs) showed increased usage of condoms, prevention of sexually transmitted infections, and reduction in unwanted pregnancies among adolescents when condoms are within school premises [22]^26^. In that study, school authorities (including teachers) permitted adolescents to possess and use condoms as part of a comprehensive school health program (CSHP) [26]. However, there is limited evidence of such interventions in Africa where cultural values shape decision-making regarding contraceptive use, including condoms [27]. Considering that some adolescents in and out of school engage in sexual relationships, a study conducted by Colleen and colleagues also mentioned that contraceptives should be among the first-line options for adolescents to prevent unwanted pregnancies [27]^25^. To address some of the challenges, Malawi should adopt the concept of health promotion in schools (HPS) that was initiated by the World Health Organization (WHO) in 1980s. This can be done by linking Education policy and Sexual Reproductive Health Right Plicy and encouraging girl child to complete her education.

Brady [30] found that young adolescents aged 10–14 years are among the ignored populations because most policies and programs target other age groups. The findings of this present study also highlighted the same challenges. No policy fully discusses issues of contraception and deciding for 10–14 aged adolescents and has been available in schools where they are mainly present. In our context, the Education policy is not consistent with the Sexual Health and Reproductive Right policy, which stipulates that young adolescents should access contraceptives within school premises [28]. This is a missed opportunity because most young adolescents are in schools. It further poses challenges for programs that aim to implement interventions and scale up contraceptive use among sexually active in-school adolescents in the country [14, 29]. The current health facility set-up seems to favor under-five children, older adolescents, and adults. Similarly, studies have revealed that sexually active young adolescents prefer special services offered to them as a motivation towards contraceptive decision-making and use. School premises was the preferred option mentioned by participants in this study [29, 30].

Further findings indicated that male adolescents lead in making decisions on contraceptive use, which is consistent with findings from Uganda [25]. If adolescents are to be champions of contraceptive use, male adolescents should be at the center of programs to improve uptake. The findings show that male adolescents have a final say on whether to use a method or not while female adolescents accept those decisions due to fear of losing the relationship [31–33]. Therefore, programs enforcing contraceptive use should emphasize empowering female adolescents in decision-making [33] which remains consistent with recommendations outlined in a brief report from DHS of 41countries that indicated empowering girls in decision making [29]. In 2011, from a brief report from DHS of 41countries indicated on empowering girls in decision making [29].

Other factors identified in this study that influence adolescents’ decisions regarding contraceptive use include environmental and personal values, which are consistent with findings from existing studies [34, 35]. As the study conceptual model suggests, social networks influence contraceptive decision making (male partners, parents, peers, and teachers), as well as environmental and policy. In addition, a meta-ethnographic study found that decision-making regarding contraceptive use among adolescents is influenced by self, partner, and family [21]. Strategies that address and incorporate these determinants with strong policy backing could contribute to increased uptake of contraceptives among adolescents and thus prevent undesirable reproductive health outcomes among this segment of the population. In addition, empowering girls in contraceptive decision making and sensitizing boys on responsible sexual behavior and respect the rights of a girl child. Given the selective nature of participants in this study, future research should consider exploring the views of young adolescents from different cultural backgrounds in the count.

### Strengthens and limitations of the study

Our study interviewed only those who attended youth-friendly health services on a day of data collection and only sexually active adolescents who used youth-friendly health services. This meant that the views of adolescents who had little access to YCs and YFHSs and were not sexually active were not considered that could affect the outcome of the study. The snapshot of data collection would not analyze perception towards contraceptives over a long period hence a bigger study that would include not active adolescents at a large scale could be able to shade more light on young adolescent perception. Lastly, some of the adolescents were not able to open up because of shyness which made it very difficult for them to express themselves, hence hiding some of the information which could have been helpful.

## Conclusion

Interventions to scale up contraceptives should cut across male and female adolescents since both are involved in decision-making regarding whether to use a method or not. Given the apparent policy contradictions regarding adolescent SRH, Malawi needs to harmonize Education and SRHR policies and to develop indicators for change to guide the implementation of programs to improve health outcomes among this segment of the population.

## Data Availability

Datasets and materials are available and can be provided upon request.

## Appendix A. Interview guide for key informants

1. What is your major roles in the organization pertaining to adolescent health?
2. In your own views, do you think teenage pregnancy is an issue in Malawi? Why?
3. Have you ever talked to your child/adolescent about sexuality and contraceptive use? Why?
4. What are the issues in relations to teenage pregnancy you would wish to share with your child/adolescent as he/she matures from childhood to adulthood?
5. What roles do you play to support adolescents to prevent teenage pregnancy?
6. Would you encourage young adolescents (10–14) to use modern family planning methods? Why?
7. Would you advocate for contraceptives in schools where these adolescents are mostly found? Why/how
8. Do you have any comment which you feel is good to share about adolescent contraceptive use?

## Appendix B. Interview guide for an adolescent

1. To explore knowledge of contraceptive use among young adolescents
2. Have you ever had relationship of the opposite sex? If so, for how long?
3. Are you currently on a relationship? What issues you like to discuss when you meet?
4. Do adolescents talk about modern family planning methods? What do they discuss?
5. Do you know someone using family planning methods? If yes, which method do they use and what about yourself?

2. To explore perception of contraceptive use among young adolescents

- Do adolescents prefer using modern family planning methods? Why?
- What would be your comment on the use modern family planning methods?
- Would adolescents like to be an advocate of modern family planning methods to sexual active young adolescents? What about yourself?

3. To explore perception of key stakeholders on contraceptives use among young adolescents

- Do adolescents discuss issues pertaining sexuality and contraceptive use with their parents/guardians? Why/How?
- Do you think parents/guardians have a role on issues of sexuality and contraceptive use? How?
- What are the things society/community would do to encourage young adolescents to use modern family planning use and prevent teenage pregnancy?

4. Explore contraceptive decision making processes on use of contraceptives in young adolescents

- What do adolescents consider important before using modern family planning methods?
- Whose opinion carry weight in terms of contraceptives use? Why?
- Who has a final say in contraceptive decision making?
- Describe how would an adolescent can access contraceptive in your setting?
- What are circumstances that affect contraceptives use among adolescents?
- Does the current system support young adolescent to use family planning methods? How?
- What type of advice would you give to the current health system to encourage more adolescents use family planning methods?

## Abbreviations

COMREC: College of Medicine Research Ethics Committee
DHIS: District Health Information System
DHS: Demographic Health Survey
DR: Doctor
FGD: Focus group discussion
HIV: Human immunodeficiency virus
LMIC: Low and middle income countries
MDHS: Malawi Demographic Health Survey
MGHI: Master of Science in Global Health Implementation
MSCE: Malawi School Certificate of Education
STI: Sexual transmitted infections
T/A: Traditional authority
TRA: Theory of reasoned action
UNC: University of North Carolina
WHO: World Health Organization
YC: Youth club
YFHS: Youth friendly health service
AGYW: Adolescents girls and young women
